# Identification and Treatment of Opioid-Associated Out-of-Hospital Cardiac Arrest in Emergency Medical Service Protocols

**DOI:** 10.1001/jamanetworkopen.2022.14351

**Published:** 2022-05-27

**Authors:** David G. Dillon, Gustavo D. Porto, Vidya Eswaran, Courtney Shay, Juan Carlos C. Montoy

**Affiliations:** 1 Department of Emergency Medicine, University of California, San Francisco; 2 Frank H. Netter MD School of Medicine at Quinnipiac University, North Haven, Connecticut; 3 National Clinician Scholars Program, Philip R. Lee Institute of Health Policy Studies, University of California, San Francisco

## Abstract

This cross-sectional study reviews emergency medical services (EMS) treatment protocols for adults presenting with cardiac arrest or overdose to evaluate whether current protocols include the consideration of opioid overdose for patients with possible out-of-hospital cardiac arrest.

## Introduction

Opioid-related deaths are the leading cause of death for individuals in the US between 25 and 64 years of age.^1^ The role of naloxone—an opioid receptor competitive antagonist—in opioid-related resuscitative emergencies continues to evolve. In a recent statement, the American Heart Association emphasized the clinical importance of early recognition of opioid overdose and identified naloxone’s potential role in treating opioid-associated out-of-hospital cardiac arrest (OA-OHCA) as one of the highest priority items for OA-OHCA research.^1^

Because OA-OHCA is often occult, and opioid-related emergencies—such as respiratory arrest with a pulse—can be confused with cardiac arrest, it is important that emergency medical services (EMS) consider opioid overdose in their evaluation of cardiac arrests. We evaluated whether current EMS protocols include the consideration of opioid overdose for patients with possible OHCA.

## Methods

In this cross-sectional study, we reviewed treatment protocols for adults presenting with cardiac arrest or overdose from 127 EMS systems covering all 50 US states between July 1 and November 23, 2021.^2^ For states without any listed protocols, we searched publicly available resources to fill this gap. After initial protocol abstraction, a random 40% subset were independently reviewed, with a data element agreement rate of 89.0%. This study was granted exemption from University of California, San Francisco, institutional review board oversight because it is not human participants research. This study followed the Strengthening the Reporting of Observational Studies in Epidemiology (STROBE) reporting guideline.

Protocols were reviewed to determine the primary outcome: whether opioid overdose was identified as a potentially reversible cause of cardiac arrest. Secondary outcomes included (1) whether naloxone was mentioned in the cardiac arrest or overdose protocols, (2) whether cardiac arrest was mentioned in the overdose protocol, and (3) whether naloxone was mentioned in protocols other than the cardiac arrest and overdose protocols. We report protocol characteristics as counts and percentages.

## Results

The most recent revision date for cardiac arrest protocols from the 127 EMS systems ranged from 2012 to 2021. A total of 108 cardiac arrest protocols (85.0%) included reversible causes of cardiac arrest; while 51 cardiac arrest protocols (40.2%) mentioned opioids, drugs, or overdose; and 29 cardiac arrest protocols (22.8%) mentioned naloxone (Table). Eight EMS systems (6.3%) specifically state that cardiac arrest is a contraindication to naloxone administration. A total of 109 overdose protocols (85.8%) mentioned naloxone, while 15 overdose protocols (11.8%) mentioned the potential for opioid-associated cardiac arrest. Naloxone was mentioned in protocols other than the cardiac arrest or overdose protocols in 84 EMS systems (66.1%).

**Table.** EMS Protocol Characteristics for Recognition and Treatment of Opioid-Associated Out-of-Hospital Cardiac Arrest

Characteristic	Protocols, No. (%) (N = 127)
Cardiac arrest protocol	
Includes reversible causes	108 (85.0)
Includes opioid, overdose, narcotic or drug	51 (40.2)
Includes naloxone	29 (22.8)
Specifically states to not give naloxone in cardiac arrest	8 (6.3)
Overdose protocol	
Includes naloxone	109 (85.8)
Includes potential for cardiac arrest due to overdose	15 (11.8)
EMS agency has other protocol that mentions use of naloxone^a^	84 (66.1)

Abbreviation: EMS, emergency medical services.

^a^ Other protocols that mention use of naloxone include, but are not limited to, altered mental status, naloxone-specific protocol for emergency medical technician or law enforcement use, pain control, and respiratory distress protocols.

## Discussion

We found that most EMS protocols do not identify opioid overdose as a potential cause of cardiac arrest. A total of 15.0% of cardiac arrest protocols did not mention any reversible causes of cardiac arrest, despite their long-standing inclusion in advanced cardiac life support guidelines.^3^

In addition, although naloxone is beneficial for patients experiencing opioid overdose who have severe respiratory depression and a pulse,^4^ mention of naloxone was notably absent from 14.2% of overdose protocols. Naloxone is likely beneficial for patients experiencing overdose with respiratory arrest and difficult-to-detect pulses and possibly beneficial for those with OA-OHCA^1^; however, these patients would not be routinely identified using most EMS cardiac arrest protocols. Whether naloxone would benefit patients experiencing cardiac arrest remains unknown, and the American Heart Association has cited this question as a high-priority topic for future research.^1^

Possible reasons for not incorporating expert consensus in EMS protocols include logistical difficulties with updating protocols, systems issues when gathering approval from regulatory agencies, or limited awareness of the role of opioids in OA-OHCA and OHCA mimics.^5,6^ Limitations of this study include the use of a convenience sample of available protocols and that published protocols may not reflect the reality of what EMS personnel practice on-scene.

Emergency medical services protocols have wide-reaching effects throughout our medical systems and communities. Given the increasing incidence of opioid overdose, improving the recognition and treatment of patients with OA-OHCA is important.
